# The use of tourniquet is useful in terms of blood loss and soft tissue damage in arthroscopic anterior cruciate ligament reconstruction: a retrospective study

**DOI:** 10.1038/s41598-023-45159-3

**Published:** 2023-10-18

**Authors:** Masaki Nagashima, Ryo Sasaki, Kentaro Tanaka, Kenichiro Takeshima

**Affiliations:** 1 Department of Orthopaedic Surgery, School of Medicine, International University of Health and Welfare, 4-3 Kōzunomori, Narita City, Chiba 286-8686 Japan; 2 Department of Orthopaedic Surgery, International University of Health and Welfare Mita Hospital, 1-4-3, Mita, Minato-Ku, Tokyo, 108-8329 Japan; 3 Department of Orthopaedic Surgery, International University of Health and Welfare Narita Hospital, 852, Hatakeda, Narita City, Chiba 286-8520 Japan

**Keywords:** Biomarkers, Outcomes research

## Abstract

Whether a tourniquet should be used for anterior cruciate ligament reconstruction (ACLR) when the operative field is secured remains controversial. Little is known about the influence of not using a tourniquet on total perioperative blood loss and soft tissue damage. The aim of this study was to compare total perioperative blood loss and soft tissue damage with and without tourniquet use during ACLR. Seventy-seven consecutive ACLRs in 76 patients were performed without tourniquet use at our hospital and enrolled in this study (T− group) between November 2018 and September 2021. The control group (T + group) comprised 55 historical ACLRs in 55 patients performed with tourniquet use at our hospital between April 2017 and September 2018. Total perioperative blood loss, calculated from the change in hemoglobin between that preoperatively and on postoperative day (POD) 1, and indicators of soft tissue damage including serum white blood cell (WBC) counts, creatine phosphokinase (CPK), and C-reactive protein (CRP) values measured on POD 1 and POD 7 were compared between groups. Total blood loss was significantly higher in the T− group (339 ± 216 mL) than in the T + group (258 ± 199 mL; *P* = 0.030). On POD 1, WBC counts were significantly higher in the T− group (9.7 ± 2.4 × 10^3^ cells/µL) than in the T + group (9.1 ± 2.5 × 10^3^ cells/µL; *P* = 0.043), CPK levels were significantly higher in the T− group (294 ± 417 U/L) than in the T + group (255 ± 88 U/L; *P* = 0.046), and CRP levels were also significantly higher in the T− group (1.40 ± 1.12 mg/dL) than in the T + group (0.91 ± 0.76 mg/dL; *P* = 0.016). No significant differences in WBC counts or CPK or CRP levels were seen between groups on POD 7. Total blood loss and soft tissue damage were significantly increased without tourniquet use during ACLR. No advantage was found for not using a tourniquet in terms of blood loss or soft tissue damage.

## Introduction

The tourniquet is widely used and useful for securing the surgical field during arthroscopic anterior cruciate ligament reconstruction (ACLR)^1^. Since the tourniquet is applied to the proximal thigh and tightened to obstruct blood flow to the affected leg, concerns have been raised regarding the potential for muscle damage at the site of direct pressure and the effects of blood stagnation and ischemia in the entire lower extremity^2,3^. In fact, perioperative complications have been reported with the use of tourniquet, including an increased incidence of postoperative deep venous thrombosis (DVT)^4–6^, quadriceps muscle weakness^7^, increased pain^8^, and nerve damage^9,10^. Using tourniquet, soft tissue damage is thought to be more severe, even if it does not lead to complication. However, a recent meta-analysis of randomized controlled trials found no significant differences in postoperative pain, operative time, or quadriceps muscle strength at 6 months after ACLR with or without a tourniquet^11^. When the operative field is secured, whether a tourniquet should be used for ACLR remains controversial^1,12,13^.

When considering surgical procedures, evaluation of perioperative blood loss and soft tissue damage is important. Postoperative bleeding from drains after ACLR is reportedly significantly less without use of a tourniquet^4,8,11,14^. However, the use of tourniquet is believed to be effective for decreasing intraoperative blood loss. Although ACLR is relatively a low invasive surgery, total perioperative blood loss should thus be evaluated, but has not yet been clarified. In addition, soft tissue damage, including inflammation, is expected to increase with tourniquet use due to direct pressure on the muscles and temporary ischemia of the lower extremities, but has not been confirmed.

The purpose of this study was to compare total perioperative blood loss and soft tissue damage with and without tourniquet use during ACLR. The hypothesis of this study was that ACLR without a tourniquet would incur greater total blood loss but would show less soft tissue damage.

## Methods

### Patients

This was a retrospective study. A total of 82 consecutive single-bundle ACLRs in 80 patients, including 10 revision surgeries, were performed without the use of tourniquet at our hospital between November 2018 and September 2021. All these cases were initially included in this study (T− group). The control group (T + group) comprised 56 historical single-bundle ACLRs in 56 patients, including 10 revision surgeries, performed with tourniquet use at our hospital between April 2017 and September 2018. Exclusion criteria were same for both groups, as follows: (1) patients requiring perioperative anticoagulation (n = 1 in each group); (2) patients who had undergone bilateral simultaneous ACLR (n = 1 in T− group); (3) patients who required additional ligament surgery or high tibial osteotomy (n = 0); 4) patients who required additional treatment for other illnesses within 7 days postoperatively (n = 2 in T− group). As a result, 77 ACLRs in 76 patients in the T− group and 55 ACLRs in 55 patients in the T + group were analyzed in this study. Since all patients were hospitalized for ≥ 1 week after surgery according to patient request, no patients were lost to follow-up or had missing data within 1 week. The current Japanese universal health insurance system allows > 7 days of hospitalization for patients undergoing ACLR. This study was performed in line with the principles of the Declaration of Helsinki, and approved by the institutional review board of International University of Health and Welfare (filing no. 5-16-55). Informed consent was waived by the ethics committee of International University of Health and Welfare.

### Surgical procedures

All ACLRs were performed by the same experienced surgeon, with the patient under general anesthesia with femoral and tibial nerve blocks. In both groups, the same ACLR procedures were performed except for the use of a tourniquet. In the T + group, the tourniquet was used only during arthroscopic procedures. At first, the graft was harvested and prepared without tourniquet use. The arthroscopic procedure was then started applying a tourniquet, and gravity infusion was used. Prior to tourniquet pressurization, the affected limb was exsanguinated, and tourniquet pressure was set at 250–280 mmHg. In the T− group, no tourniquet was applied, and an irrigation pump (DYONICS 25; Smith & Nephew, Andover, MA) was used, with pressure initially set at 40 mmHg but adjusted according to bleeding conditions to a maximum of 80 mmHg. To control bleeding, a radiofrequency device (QUANTUM 2; Smith & Nephew) was also used intraoperatively in both groups. Anterior cruciate ligaments were reconstructed using single-bundle hamstring autografts. For graft preparation, a commercially available polyester tape (Leeds-Keio artificial ligament; Neoligament, Leeds, UK) was connected at the distal end of the graft, and an Endobutton CL BTB or Ultra Button (Smith & Nephew) was attached at the proximal end of the graft. The graft was introduced through each tibial tunnel and the femoral tunnel, all of which were made using the transportal technique. The diameter of the bone tunnel was matched to the diameter of the hamstring autografts, at 8–10 mm. The graft was fixed using the Endobutton or Ultra Button on the femoral side and two staples on the tibial side. In revision ACLR, the hamstring autograft was harvested from the contralateral side, and ACLR was performed in the same procedure as the primary ACLR. Meniscus tears that were repairable were repaired using an all-inside technique with FAST-FIX360 (Smith & Nephew) or an outside-in technique. Middle-to-posterior meniscus injuries were repaired with the all-inside technique, and anterior meniscus injuries were repaired with the outside-in technique. As intraoperative infusion, acetated Ringer's solution was used intravenously. A closed-suction drain was kept in the knee for 24 h.

### Postoperative care

Postoperatively, the knee was immobilized with a brace for one day for patients without meniscus repair, and for five days for patients with meniscus repair. After knee immobilization, range-of-motion exercises and muscle strengthening were started. Full weight-bearing with a hinged knee brace was allowed from POD 7. For DVT prophylaxis, intermittent pneumatic compression for foot was applied to the contralateral limb during ACLR. Although no pharmacological prophylaxis was administered, intermittent pneumatic compressions for bilateral feet were used for one day, and compression stockings that covered the entirety of both legs were used for 7 days following ACLR.

A 1000 ml acetated Ringer's solution was given intravenously during 12 h postoperatively. For postoperative pain control, a continuous femoral nerve block delivering 0.2% ropivacaine at 4 mL/h was used for 24 h, and celecoxib (200 mg, twice daily) was administered orally for 1 week after ACLR. If pain control proved inadequate, a diclofenac sodium suppository (50 mg) and/or intravenous acetaminophen (1000 mg) was used, as appropriate.

### Outcome measurements

The primary outcomes of this study were total perioperative blood loss and soft tissue damage. Postoperative pain, which might be affected by tourniquet use, was defined as a secondary outcome in this study.

Total blood loss was computed from estimated preoperative patient blood volume (PBV) and the change in hemoglobin (Hb) between that preoperatively and that on POD 1. Preoperative blood test was conducted on any day within the month prior to surgery. PBV was computed according the formula: PBV (L) = (k1 × height [m]^3^) + k2 × body weight [kg]) + k3. In this formula, k1 was 0.3669 for men and 0.3561 for women, k2 was 0.03219 for men and 0.03308 for women, and k3 was 0.6041 for men and 0.1833 for women^15^. The drop in Hb from that preoperatively to that on POD 1 was defined as ΔHb (ΔHb = preoperative Hb − POD1 Hb). We calculated total blood loss according the formula: total blood loss (mL) = 1000 × PBV × ΔHb/preoperative Hb. As indicators of soft tissue damage and inflammation, serum white blood cell (WBC) count and creatine phosphokinase (CPK), and C-reactive protein (CRP) levels were measured preoperatively and on POD 1 and POD 7. Postoperative intensity of pain in the affected knee was assessed using a numerical rating scale (NRS, 0–10). NRSs were assessed at 3 h after ACLR and at 10:00 AM on POD 1, POD 2, POD 3, and POD 7.

### Data collection

Preoperative clinical and surgical data including age, sex, body mass index (BMI), Lysholm score, time interval from injury to ACLR (< 3 months or ≥ 3 months), primary or revision ACLR, presence or absence of meniscus repair, intraoperative intravenous infusion volume, operative time, and tourniquet time were recorded. As postoperative data, blood loss from the drain was recorded.

### Statistical analysis

In addition to the primary and secondary outcomes, recorded data were compared between groups using Student’s *t*-test, the Mann–Whitney *U* test, and the χ^2^ test. Values of *P* < 0.05 were considered significant. These statistical analyses were performed using BellCurve for Excel (Social Survey Research Information, Tokyo, Japan). On the sample size calculation, we used the following parameters: an overall 2-sided significance level of 0.05, 80% power, a between-group difference for the change in mean perioperative calculated blood loss score of 100 mL, and a within-group standard deviation of 150 mL. Using these parameters, 36 knees were estimated to be needed per group. The sample size calculation was performed using R version 4.0.3. (R Foundation for Statistical Computing, Vienna, Austria).

## Results

For preoperative clinical and surgical data, no significant difference was seen between groups (Table 1). No patients had issues with visualization during surgery and no complications associated with prolonged high pump pressure were seen in the T− group. Among patients who required meniscus repair, the number of meniscus sutures was 2.3 ± 1.2 in the T + group and 3.2 ± 1.7 in the T− group (*P* = 0.059). Although no significant differences in Hb were seen preoperatively or on POD 1, ΔHb and total blood loss were significantly higher in the T− group. Conversely, blood loss from the drain at 24 h postoperatively was significantly higher in the T + group (Table 2).

**Table 1:** Preoperative clinical and surgical demographics.

		T + group (n = 55)	T− group (n = 77)	*P*
Age (years)		30.5 ± 15.1	29.4 ± 13.7	0.662
Sex	Female	28	40	0.746
	Male	27	37	
BMI (kg/m^2^)		22.6 ± 2.5	23.3 ± 3.6	0.199
WBC (× 10^3^ cells/µL)		6.4 ± 1.8	6.4 ± 1.5	0.858
CPK (U/L)		160 ± 206	136 ± 147	0.995
CRP (mg/dL)		0.07 ± 0.14	0.10 ± 0.12	0.198
Lysholm score		71.1 ± 13.6	67.1 ± 15.2	0.124
Time interval from injury to ACLR	< 3 M	22	37	0.359
	≥ 3 M	33	40	
ACLR	Primary	45	67	0.412
	Revision	10	10	
Meniscus repair	(+)	20	41	0.055
	(−)	35	36	
Intraoperative intravenous infusion volume (ml)		757 ± 203	734 ± 185	0.498
Operative time (min)		80.8 ± 18.7	79.9 ± 15.5	0.748
Tourniquet time (min)		68.5 ± 14.7	0	NA

Data are reported as mean ± standard deviation unless otherwise indicated.

*BMI* body mass index, *WBC* white blood cell, *CPK* creatine phosphokinase, *CRP* C-reactive protein, *ACLR* anterior cruciate ligament reconstruction, *NA* not applicable, *T+*, with tourniquet use, *T−*, without tourniquet use.

**Table 2:** Perioperative Hb levels and blood loss.

	T + group (n = 55)	T− group (n = 77)	*P*
Preoperative Hb (g/dL)	14.3 ± 1.2	14.6 ± 1.3	0.159
Hb on POD 1 (g/dL)	13.4 ± 1.4	13.4 ± 1.3	0.826
ΔHb (g/dL)	0.9 ± 0.7	1.2 ± 0.7	0.040
Total blood loss (mL)	258 ± 199	339 ± 216	0.030
Blood loss from drain (mL)	202 ± 77	152 ± 64	< 0.001

Data are reported as mean ± standard deviation unless otherwise indicated.

*Hb* hemoglobin, *POD* postoperative day, *T+*, with tourniquet use, *T−*, without tourniquet use.

**Figure 1:**
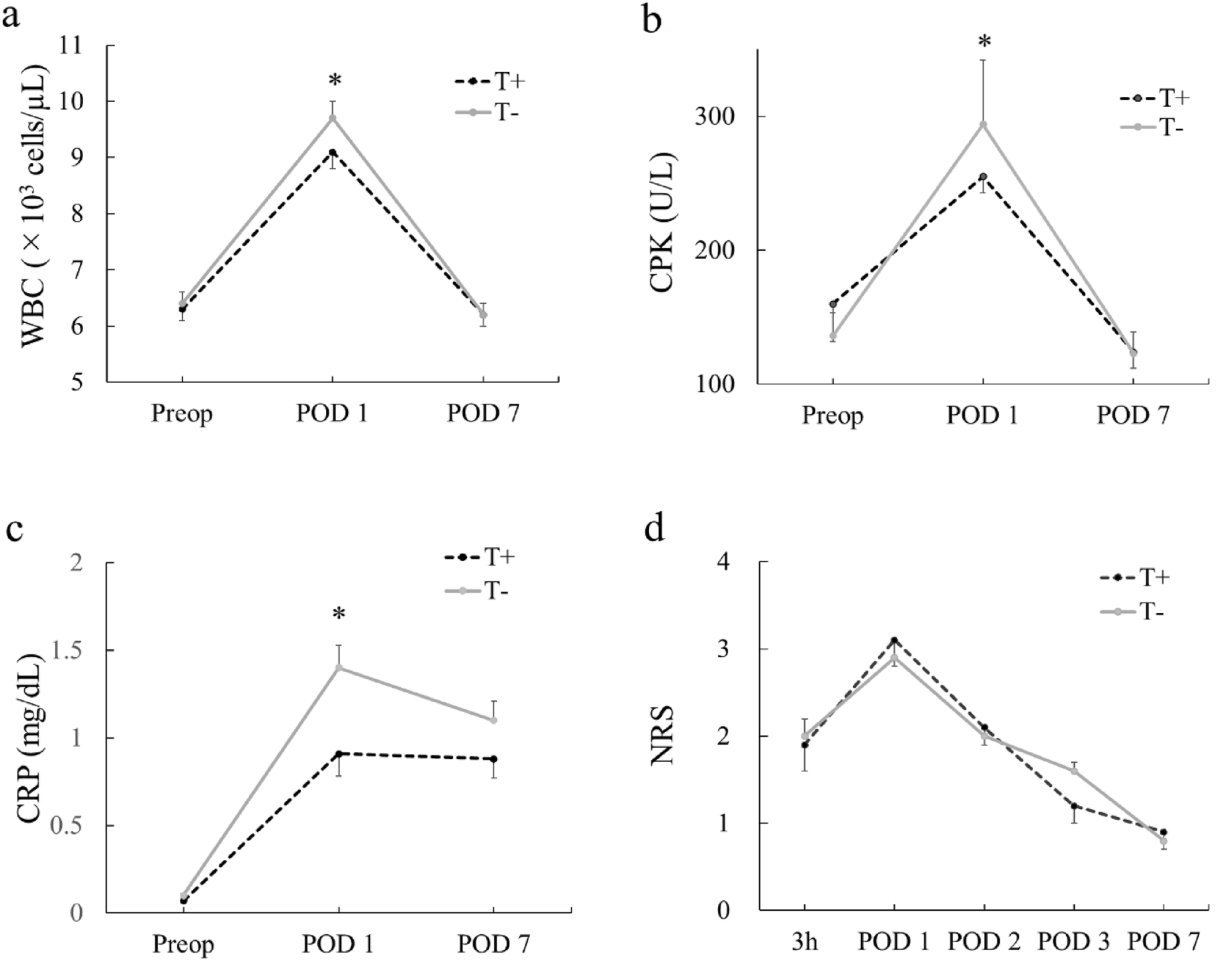
Comparisons between groups. (**a**) Serum white blood cell (WBC) counts. No significant differences were found between groups preoperatively or on postoperative day (POD) 7, but were evident on POD 1. WBC counts were significantly higher in the T− group than in the T + group on POD 1. *Significant difference (*P* < 0.05) between groups. (**b**) Serum creatine phosphokinase (CPK) levels. No significant differences were found between groups preoperatively or on POD 7, but were evident on POD 1. CPK levels were significantly higher in the T− group than in the T + group on POD 1. *Significant difference (*P* < 0.05) between groups. (**c**) Serum C-reactive protein (CRP) levels. No significant differences were found between groups preoperatively or on POD 7, but were evident on POD 1. CRP levels were significantly higher in the T− group than in the T + group on POD 1. *Significant difference (*P* < 0.05) between groups. (**d**) Postoperative numerical rating scale (NRS). No significant differences in postoperative pain of the affected knee as assessed by NRS score (0–10) were found between groups at any time of the survey.

WBC counts showed no significant difference between groups preoperatively or on POD 7, but on POD 1, WBC counts were significantly higher in the T− group (9.7 ± 2.4 × 10^3^ cells/µL) than in the T + group (9.1 ± 2.5 × 10^3^ cells/µL; *P* = 0.043) (Fig. 1a). In CPK levels, no significant difference between groups was found preoperatively or on POD 7, but CPK levels on POD 1 were significantly higher in the T− group (294 ± 417 U/L) than in the T + group (255 ± 88 U/L; *P* = 0.046) (Fig. 1b). CRP levels showed no significant difference between groups preoperatively or on POD 7, but CRP levels on POD 1 were significantly higher in the T− group (1.40 ± 1.12 mg/dL) than in the T + group (0.91 ± 0.76 mg/dL; *P* = 0.016) (Fig. 1c). No significant differences in postoperative pain of the affected knee as assessed by NRS were identified between groups at any time of the survey (Fig. 1d).

## Discussion

The most important findings of the present study were that ACLR without tourniquet use resulted in a significantly increased total blood loss and soft tissue damage, but no difference in postoperative knee pain was evident between groups. Conversely, postoperative blood loss from the drain was significantly higher in ACLR with tourniquet use. The present results for blood loss support the hypothesis that total blood loss is increased without the use of a tourniquet during ACLR. However, the results for soft tissue damage were counter to the hypothesis, instead showing an increase without the use of a tourniquet during ACLR. To the best of our knowledge, this represents the first report to demonstrate that not using a tourniquet offers no advantages for limiting total perioperative blood loss or soft tissue damage in ACLR.

Postoperative blood loss from the drain is reportedly significantly lower when a tourniquet is not used during ACLR^4,8,11,14^, and the results of the present study support such findings. This was thought to be a result of the bleeding site that could be identified and hemostasis that could be achieved by radiofrequency devices in ACLR without the use of a tourniquet. However, total perioperative blood loss was significantly higher without tourniquet use in this study. The difference between total blood loss and postoperative blood loss from the drain that indicates mainly intraoperative bleeding was higher in ACLR without tourniquet use. Without tourniquet use, bleeding persisted intraoperatively even with hemostasis, and total blood loss was considered to be significantly higher.

Serum WBC counts and CPK and CRP levels were used as indicators of soft tissue damage in the current study. Although these values were expected to increase with tourniquet use due to direct compression of the muscles and ischemia in the entire lower extremity, both were rather significantly elevated without tourniquet use. Similar results for CPK and CRP have also been reported in total knee arthroplasty^16^. The surgical procedures of ACLR were almost identical between the two groups. But without tourniquet use, more hemostatic maneuvers involving the synovial tissue were required to secure the surgical field with a radiofrequency device, even though they did not make a significant difference in operative time. CPK is found in synovial tissue as well as muscle^17^. Inflammatory cell infiltration as well as fusion of collagen and pyknosis of fibroblasts are reportedly observed at the site of soft tissue damage caused by a radiofrequency device^18^. During hemostasis, some synovial tissue may be damaged, causing CPK elevation and leading to synovitis, in turn causing elevations in WBC and CRP. Arciero et al. compared serum CPK after ACLR with and without tourniquet use and reported no significant differences between groups, differing from our results. They did not provide details of hemostatic procedures, and mean operative time was 128 min in the tourniquet group and 137 min in the no-tourniquet group. This was longer than our operative time and may have affected the results^19^.

For postoperative pain, Reda et al. evaluated pain using the visual analogue scale, finding significantly higher scores for tourniquet use up to 10 h after ACLR^8^. However, no significant difference in postoperative pain was evident between groups in our study. Postoperative pain is believed to be influenced by postoperative pain control practices. In the present study, the use of 24-h continuous femoral nerve block may have affected the results. Nakayama et al. performed ACLR under general anesthesia and femoral nerve block, and reported no differences in postoperative pain with or without tourniquet use^14^. In addition, Hooper et al. used intravenous patient-controlled analgesia for postoperative pain management and reported no difference in postoperative pain or morphine consumption with or without tourniquet use^1^.

This study had some limitations. First, the number of patients was small and historical control was used. And the study did not use a randomized controlled design. Second, postoperative development of hemarthrosis is a concern in ACLR, but was not evaluated in this study. Third, several other indicators of soft tissue damage are available besides than WBC counts and CPK and CRP levels, but were not investigated^20,21^. Fourth, the amount of diclofenac sodium suppository and/or intravenous acetaminophen used for inadequate postoperative pain control was not investigated. No significant differences in postoperative pain were apparent between groups, but differences in amounts of those medications used may have been present. Fifth, there was a tendency for more cases requiring meniscus sutures in the T− group, which might have affected the results. Sixth, the Hb level on POD 1 does not reflect only blood loss, but is affected by factors such as the amount of perioperative intravenous infusions.

## Conclusions

Although total blood loss and soft tissue damage were significantly increased without tourniquet use during ACLR, no difference in postoperative knee pain was identified. Use of a tourniquet was reported to carry a risk of complications, but no advantage was found for not using a tourniquet in terms of blood loss or soft tissue damage.

## Data Availability

The datasets used and/or analysed during the current study are available from the corresponding author on reasonable request.
